# The contralateral progression in a cohort of Chinese adult patients with unilateral moyamoya disease after revascularization: a single-center long-term retrospective study

**DOI:** 10.1007/s00701-022-05153-6

**Published:** 2022-03-26

**Authors:** Xiao Tian, Miao Hu, Jianjian Zhang

**Affiliations:** Department of Neurosurgery, Zhongnan Hospital of Wuhan University, Donghu Road 169, Wuhan, 430071 China

**Keywords:** Adult, Chinese, Moyamoya disease, Progression, Unilateral

## Abstract

**Background:**

Moyamoya disease (MMD) is a chronic progressive cerebrovascular disease mainly existing in the Asian population, which can be divided into unilateral and bilateral types. Contralateral progression has been reported in pediatric patients with unilateral MMD, while large series about contralateral progression in Chinese adult patients were rare. The goal of this study is to elucidate the clinical features and incidence of contralateral progression in Chinese MMD adult patients.

**Methods:**

One hundred one Chinese adult patients with unilateral MMD who received surgery treatments between January 2015 and January 2017 in our hospital were enrolled in this study. This study contained 89 patients. Digital subtraction angiography was performed in all patients for initial diagnosis, and magnetic resonance angiography was repeated 6 months from the initial operation and then annually. Clinical characteristics, contralateral progression, and risk factors were studied. Previous related studies were also reviewed and meta-analyzed.

**Results:**

Of these 89 patients, contralateral progression was identified in 8 patients (9.0%) within a median follow-up period of 63 months, which was lower than that in previous studies (25.9%). Single-factor analysis and multivariate analysis did not reveal significant risk factors related to the contralateral progression.

**Conclusion:**

The progress rate in this cohort of Chinese adult patients with unilateral MMD after revascularization was 9.0%, which indicates that some of the unilateral MMD were an early form of bilateral MMD rather than a separate condition.

Trial registration.

This work was approved by the Medical Ethics Committee of Zhongnan Hospital of Wuhan University (approval number: Kelun-2017005).

**Supplementary Information:**

The online version contains supplementary material available at 10.1007/s00701-022-05153-6.

## Background

Moyamoya disease (MMD) is a chronic cerebrovascular occlusive disease that is characterized by developing stenosis or occlusion at the distal internal carotid artery (ICA) and/or the initial anterior cerebral artery (ACA) and middle cerebral artery (MCA), complemented with the formation of intensive moyamoya-like vessels [23]. The typical MMD was defined as moyamoya-specific angiographic changes in bilateral hemispheres [6]. However, MMD can be considered “unilateral” if typical MMD angiographic changes are observed on one side, while normal angiography on the contralateral side [15, 17]. The annual incidence of bilateral MMD and unilateral MMD is respectively 1.13/100,000 and 0.23/100,000 in Japan [7]. The age distribution of MMD is significantly bimodal, with a moderate peak in the first decade of life and a major peak at 35–45 years of age [3]. This brings about essential questions: Is adult MMD a continuum of pediatric MMD? Does unilateral MMD, which is more common in adults, progress to bilateral MMD [15]? Previous studies had mainly focused on the contralateral progression changes in pediatric MMD patients [20, 21], while large series concerning the progression from adult unilateral MMD to bilateral lesions, especially in those with Chinese ethnic background, were rare [14, 16]. Up to now, there were only 14 clinical studies regarding the unilateral MMD processed to bilateral lesions [8, 9, 11–16, 18–21, 24, 26] (6 from South Korea, 6 from Japan, 1 from the USA, 1 from China). The sample size of unilateral MMD patients in all studies was relatively small (< 70), except the latest two (> 100) [18, 26].

The present study described the clinical features in a cohort of Chinese adult patients with unilateral MMD after revascularization from our hospital and tried to clarify the characteristics of contralateral progression in these patients.

## Methods

### Patient selection and data collection

This retrospective study included all consecutively treated adult (> 18 years) individuals who visited our hospital between January 2015 and January 2017. The unilateral MMD was diagnosed via the digital subtraction angiography (DSA) in accordance with the following criteria: (1) stenosis/occlusion changes at the end of ICA or the proximal ACA or MCA on one cerebral hemisphere (which was identified as the original side); (2) moyamoya-like vascular networks in arterial areas near the occlusive or stenotic lesions; (3) absence of arterial stenosis or occlusion on the contralateral side; and (4) exclusion of conditions associated with moyamoya syndrome (MMS) such as intracranial atherosclerosis, systemic vasculitis, Down syndrome, acute stroke, hyperthyroidism, meningitis, neurofibromatosis, prior skull‐base radiation therapy, leptospiral infection, or autoimmune disease. The angiographic stage was identified according to the Suzuki Grading System [23]. The single-photon emission computed tomography (SPECT) was adopted to check the perfusion status. The indication to surgery included patients with symptomatic MMD (ischemic or hemorrhagic) with apparent hemodynamic compromise. [25].

At 6 months after the initial surgery, magnetic resonance angiography (MRA) was repeated and then repeated annually if the patient remained asymptomatic. The contralateral progression was defined as the detection of newly developed stenosis at the terminal ICA or the proximal ACA or MCA or posterior cerebral artery (PCA) with an MRA steno-occlusive change in the previously intact hemisphere during the follow-up period [26]. The DSA was performed to confirm before the contralateral revascularization. Atherosclerosis or other etiologies which can also cause intracranial vessel stenosis were excluded during the follow-up period.

One hundred one unilateral MMD adult patients were initially identified; all of them were treated with the standard superficial temporal artery (STA)-MCA bypass combined with encephalo-duro-myo-synangiosis (EDMS) revascularization by 2 surgeons (J.Z. or J.C.) on the original side according to the published procedures [25]. Among them, 10 patients were excluded for the lack of necessary follow-up information, and 2 patients were excluded for the follow-up duration of less than 36 months. Thus, 89 unilateral MMD patients were included in this study.

The baseline characteristics including age, sex, associated disease, and family history were collected from the electric medical record system. The onset of MMD was divided into 4 types: transient ischemic attack (TIA), ischemic (infarction, frequent TIA), hemorrhagic (intracerebral hemorrhage), and other (headache, motor dysfunction, etc.).

### Search strategy and study identification

In order to analyze the development of contralateral MMD more comprehensively, a comprehensive search was performed to identify studies regarding the unilateral MMD processed to bilateral lesions. A literature search was performed in PubMed, Embase, and Ovid via all the possible combinations of “moyamoya disease,” “progression,” “unilateral,” and “bilateral” up to March 20, 2020. We only included studies satisfied with the following criteria: (1) patients with MMD diagnosis confirmed by radiological and clinical criteria, (2) the sample size of unilateral MMD patients > 5, (3) include the description of the contralateral progression, and (4) the necessary follow-up information is available. We excluded those studies without clearly distinguishing between MMD and MMS, as well as studies presented by languages other than English. References in identified articles were also manually screened.

Two reviewers independently reviewed full texts of all identified studies and independently extracted data from the included studies. Discrepancies between the two reviewers were resolved by discussion.

### Statistical analysis

SPSS (version 26.0; SPSS Inc, Chicago, IL) was used for all statistical analyses. Data were reported as numbers (percentage) or medians (interquartile ranges, IQR). The Mann–Whitney U test was used to compare continuous variables that do not obey normal distribution, and categorical variables were compared using the chi-square test. Logistic regression analysis was used to detect risk factors related to contralateral progression. The contralateral progress rate was transformed with the Freeman-Tukey variant of arcsine square prior to statistical pooling. For every analysis, *P* < 0.05 was considered statistically significant.

## Results

### Clinical results

Among the 89 Chinese adult patients with unilateral MMD (median age: 47 years, range: 31–54 years, 53 females [59.6%] and 36 males [40.6%]), original moyamoya-angiographic change was observed in 52 right hemispheres (58.4%) and 37 left hemispheres (41.6%). The angiographic Suzuki stages on the original side were mainly III (41.6%) and IV (37.1%). Clinical onsets: 34 cases were hemorrhagic (38.2%), 28 cases were ischemic (31.5%), 22 cases were TIA, and 5 were other symptoms (5.6%). Associated diseases were observed in 29 patients (32.6%), with 12 hypertensions (13.5%), 6 aneurysms (6.7%), 3 diabetes (3.4%), and 8 other diseases (9.0%) like hyperthyroidism, pneumonia, and hepatitis B. Nine patients (10.1%) had a family history of MMD. All patients received STA-MCA bypass surgery combined with EDMS revascularization. The follow-up duration median was 63 months, ranging from 48 to 78 months. All baseline characteristics were summarized in Table 1.

**Table 1:** Baseline characteristics of included cases with unilateral moyamoya disease

	All patients (*n* = 89)
Age (year) (medians (IQR))	47 (31–54)
Sex (*n* (%))	
Female	53 (59.6)
Male	36 (40.6)
Original side (*n* (%))	
Right	52 (58.4)
Left	37 (41.6)
Suzuki stage on the original side (*n* (%))	
II	8 (9.0)
III	37 (41.6)
IV	33 (37.1)
V	10 (11.2)
VI	1 (1.1)
Onset on the original side (*n* (%))	
Hemorrhagic	34 (38.2)
Ischemic	28 (31.5)
TIA	22 (24.7)
Other	5 (5.6)
Associated disease (*n* (%))	29 (32.6)
Hypertension	12 (13.5)
Aneurysm	6 (6.7)
Diabetes	3 (3.4)
Other	8 (9.0)
Family history (*n* (%))	9 (10.1)
Follow-up duration (months) (medians (IQR))	63 (48–78)

Abbreviation: *IQR* interquartile ranges, *TIA* transient ischemic attack

### Contralateral progression

**Fig. 1:**
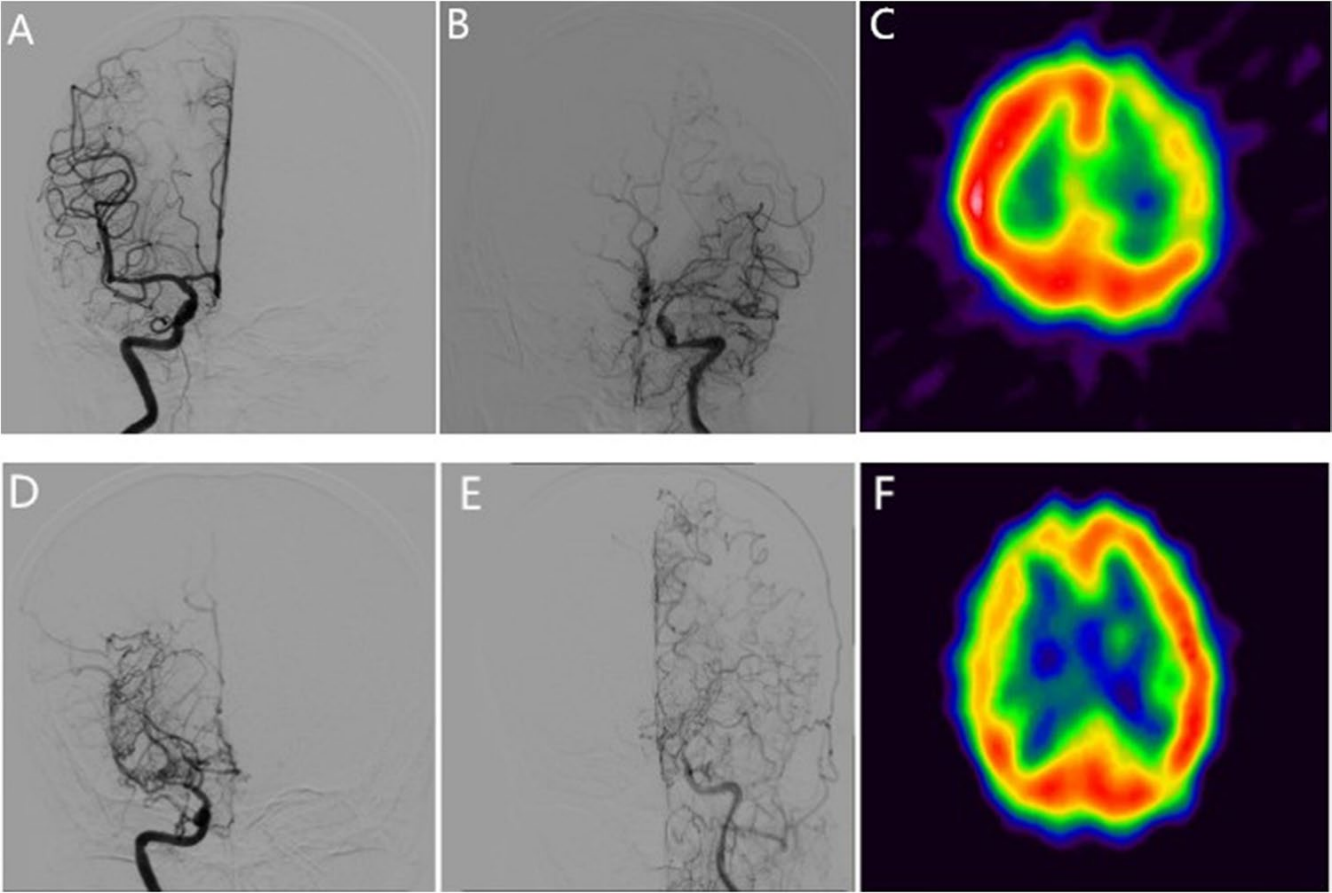
DSA from a representative case of a female patient showed that the right side was normal (**A**), while moyamoya angiographical changes were at the left side (**B**). The SPECT showed hypoperfusion on the left side (**C**). Thus, the patient received left bypass surgical treatment. Three years later, the DSA found an excessive contralateral progress on the right side (**D**) and confirmed the patency of the bypass on the left (**E**), and the SPECT showed hypoperfusion on the right side (**F**)

During the follow-up period, the contralateral progression was detected in 8 patients (representative case in Fig. 1). The progress rate was 9.0% (8/89). Among them, 5 patients were female, and 3 were male. The progression occurred on 5 left hemispheres and 3 right hemispheres. The detailed baseline demographics and medical profiles of these 8 patients are listed in Table 2. All patients had received combined bypass surgery after contralateral progression. No perioperative complication was detected.

**Table 2:** Clinical characteristics of 8 cases with contralateral procession

Patient	Age (year)	Sex	Follow-up duration (months)	Original side (Suzuki stage)	Onset	Family history	Associated disease	Procession side (Suzuki stage)	Symptom on procession side	Surgery on procession side
1	31	M	54	L (5)	Ischemic	-	-	R (3)	Ischemic	Combined
2	47	F	56	L (4)	TIA	-	-	R (2)	TIA	Combined
3	49	F	78	L (4)	TIA	-	-	R (2)	TIA	Combined
4	53	M	75	L (4)	Ischemic	-	Hypertension	R (3)	TIA	Combined
5	46	F	76	L (3)	TIA	-	-	R (3)	TIA	Combined
6	39	F	75	R (4)	TIA	Yes	-	L (3)	Ischemic	Combined
7	38	M	62	R (3)	Hemorrhagic	-	-	L (3)	Hemorrhagic	Combined
8	39	F	64	R (2)	TIA	Yes	Aneurysm	L (2)	TIA	Combined

We then divided all patients into “progression group” (8 patients) and “non-progression group” (81 patients) according to whether there was a contralateral progression. In order to determine if there was a significant difference between these two groups or any risk factor related to the progression of the initial unaffected side, single-factor analysis (Table 3) and multivariate analysis (Table 4) were performed. However, no statically significant difference was found in terms of age, sex, original side (including hemisphere, stage, and onset), associated disease, family history, or follow-up duration.

**Table 3:** Comparison between the non-progression group and progression group

	Non-progression group (*n* = 81)	Progression group (*n* = 8)	*P* value
Age (year) (medians (IQR))	48.5 (34–54)	42.5 (31–53)	0.15
Sex (*n* (%))			0.86
Female	48 (59.3)	5 (62.5)	
Male	33 (40.7)	3 (37.5)	
Original side (*n* (%))			0.21
Right	49 (60.5)	3 (37.5)	
Left	32 (39.5)	5 (62.5)	
Suzuki stage on the original side (*n* (%))			0.88
II	7 (8.6)	1 (12.5)	
III	35 (43.2)	2 (25.0)	
IV	29 (35.8)	4 (50.0)	
V	9 (11.1)	1 (12.5)	
VI	1 (1.2)	0	
Onset on the original side (*n* (%))			0.07
Hemorrhagic	33 (40.7)	1 (12.50)	
Ischemic	26 (32.1)	2 (25.00)	
TIA	17 (21.0)	5 (62.50)	
Other	6 (6.2)	2 (25.0%)	
Associated disease (*n* (%))	27 (33.3)	4 (50.0)	0.90
Hypertension	11 (13.6)	1 (12.5)	
Aneurysm	5 (6.2)	1 (12.5)	
Diabetes	3 (3.7)	0	
Other	8 (9.9)	0	
Family history (*n* (%))	7 (8.6)	2 (25.0)	0.14
Follow-up duration (months) (medians (IQR))	63 (48–78)	67.5 (54–78)	0.36

Abbreviation: *IQR* interquartile ranges, *TIA* transient ischemic attack

*P* < 0.05 was considered statistically significant difference

**Table 4:** Logistic regression analysis of risk factors related to contralateral progression

	OR	95% CI	*P* value
Age (year)	0.95	0.88–1.02	0.16
Sex	1.40	0.22–8.97	0.73
Original side	0.18	0.02–1.53	0.12
Suzuki stage on original side	1.30	0.51–3.36	0.58
Onset on original side	1.75	0.60–5.13	0.31
Associated disease	0.49	0.03–8.06	0.62
Family history	5.15	0.37–71.9	0.22
Follow-up duration (months)	0.97	0.87–1.08	0.58

Abbreviation: *OR* odds ratio, *CI* confidence interval

*P* < 0.05 was considered statistically significant difference

## Discussion

Unilateral MMD had been considered “probable” MMD to distinguish it from the “defined” bilateral MMD. To avoid possible misunderstandings, the term “unilateral MMD” had been proposed in the guideline for MMD in 2011 [17]. Same genetic defects such as the *RNF213* mutation might result in different phenotypes of unilateral or bilateral MMD [2]. However, up to now, it is still not clear whether there is a certain degree of difference in etiology and natural history between the unilateral and the usual bilateral MMD. Even so, the unilateral MMD might be a suitable model to examine the pathogenetic process of MMD, because the bilateral MMD exhibits complex and ambiguous pathological findings that do not allow identification of cause versus effect [18]. In order to accurately analyze the progression of the unilateral MMD, MMS was excluded in our study because the MMS-associated diseases such as sickle cell disease, Graves’ disease (GD), and Down syndrome [1, 5] might have positive or negative effects on the angiographic and symptomatic progression. For example, Chen et al. [5] observed that the disease progression was more frequent in MMS patients with GD than that in patients without GD during the follow-up period, especially in unilateral disease.

Unilateral MMD was reported more common in females [7]. In our study, the female–male ratio was approximately 1.5:1, which was consistent with previous studies. Unilateral MMD was frequently reported to develop into bilateral lesions in pediatric patients [9]. The similar phenomenon was rarely reported in adult patients [8], and the progress rate of unilateral MMD to bilateral MMD was varied in previous studies. According to our literature review, 2 studies [15, 16] only focused on adult patients (progress rates were 17.8% and 36.4%, respectively), while 6 studies [9, 11, 20, 21, 24, 26] only focused on pediatric patients. The median progress rate in previous studies was 29.01% (ranges from 0 to 100%). In our study, all included samples were adult unilateral MMD patients. The progress rate was 9.0%, which was lower than previous reports. The reason might be due to the relatively short follow-up duration in our study. In previous studies, the contralateral progression could take 7 to 8 years after initial diagnosis [16]. Therefore, the period between primary diagnosis and disease progression was notably extended in some cases, and long-term follow-up would be crucial for the detection of the complete progression of unilateral MMD. The different definitions of “unilateral MMD” in various studies might also have an impact on the progression rate. For example, some researchers not only considered patients with normal angiographic findings of the contralateral hemisphere as “unilateral,” but also those with mild abnormalities in the contralateral ICA, MCA, or ACA [14, 22], while some others only included patients with a normal angiographic appearance in the contralateral side [24]. In the present study, patients who originally showed contralateral cerebrovascular stenosis on DSA, even very slight, were judged as bilateral MMD and excluded in the patient selection stage. Therefore, during the limited follow-up period, we might observe fewer progressive patients. In addition, the Chinese ethnic background might also contribute to the lower progress rate, since almost all previous reaches were conducted within the Japanese or South Korean population who seem to have more excessive prevalence and incidence of MMD [25]. Indeed, the progress rate in a cohort of Chinese adults and pediatric MMD patients reported by Zhang et al. [26] in 2016 was also relatively lower (16.5%) than most studies.

Several studies have reported various risk factors regarding the contralateral progression, such as the age at diagnosis [24], the contralateral equivocal/mild angiographic stenosis change [14], and the familial history [20]. In our study, although we compared several parameters between the progression group and the non-progression group, no significant risk factor had been found. The lack of significance might be due to the small numbers analyzed, because statistical analysis for risk factors on progression in only 8 patients with progression lacks reference significance; however, other possible explanations are also suggested. On the one hand, there existed contradictory results regarding those reported risk factors among the previous studies. For example, Kelly et al. [14] observed that the initial contralateral ACA angiographic defect represented an indicator of contralateral progression, while Yeon et al. [24] reported no association between the visibility of the contralateral A1 segment and the contralateral progression. On the other hand, the mechanisms of unilateral progression to bilateral MMD might involve genetic and structural complexity such as the dysregulation of endothelial remodeling and vascular cells [4], which need more professional detection methods to explore. Recently, Lee et al. [18] used the time-of-flight (TOF) sequence from brain magnetic resonance imaging (MRI) to measure the endothelial shear stress around the normally appearing terminal ICA and found that the endothelial shear stress was an independent indicator to predict the contralateral progression of unilateral MMD. Another factor, the degree of involvement of the posterior circulation, may also represent a possible “biomarker” for aggression of disease progression [10]. However, there was no posterior circulation involvement in our 8 patients with contralateral progression.

The exploration of how unilateral MMD develops to bilateral MMD could provide indirect but indispensable evidence to understand the MMD as an entirety and progressive diseases. Clinically, the identification of unilateral patients liable to develop bilateral disease also would be of considerable clinical benefit [24]. Understanding the expected process of progression, accompanied with the understanding of specialized risk factors related to the development of the contralateral lesions, would give neurosurgeons a more precise clue to design personalized follow-up strategies [26]. For the unaffected side, instead of prophylactic contralateral revascularization, careful and long-term follow-up was more necessary for the early detection of contralateral progression, so as to timely determine surgical revascularization. Early detection and prevention of ischemic or hemorrhagic stroke were crucial for improving the prognosis of patients.

The present study has limitations. First, as mentioned above, the follow-up duration in this study was relatively short. The mean follow-up period for the whole group was just over 5.25 years. Longer follow-up time might reveal more patients with progress. And in order to reduce the DSA radiation received by patients and the cost of reexamination, we used MRA as the way of reexamination after surgery. Although this is an acceptable way according to many published studies, we do agree that this approach may ignore subtle changes that would have been identified via routine DSA. This may explain our low incidence of progression. Second, since this is a retrospective analysis of a cohort of Chinese adults with unilateral MMD, the results were limited by selection bias. Third, we did not perform hemodynamic comparison in these two groups such as cerebral blood flow, oxygen extraction fraction, or cerebrovascular reserve, although which had not been found significantly associated with contralateral progression in previous studies. And we also did not perform additional tests such as *RNF213* gene subtype or vessel wall MRI data, which may further explain potential mechanisms of the contralateral progression. Fourth, the patient population in this study was relatively small. Since the patients with unilateral MMD only account for roughly 17% in all MMD patients, it is really difficult to have much more qualified patients in one hospital, and this may also explain why previous studies regarding this topic almost had a relatively small patient population.

## Conclusion

We found a 9.0% progression rate of unilateral MMD after revascularization develops to bilateral MMD in a cohort of Chinese adult MMD patients. Our results indicated that some of the unilateral MMD were early forms of bilateral MMD rather than a separate condition; therefore, neurosurgeons ought to notice this potential change during the postoperative follow-up period.

## Supplementary Information

Below is the link to the electronic supplementary material.

Supplementary file1 (PDF 205 KB)

## Data Availability

The data that support the findings of this study are available from the corresponding author upon reasonable request.

## Abbreviations

MMD: Moyamoya disease
ICA: Internal carotid artery
ACA: Anterior cerebral artery
MCA: Middle cerebral artery
DSA: Digital subtraction angiography
MRA: Magnetic resonance angiography
MMS: Moyamoya syndrome
STA: Superficial temporal artery
EDMS: Encephalo-duro-myo-synangiosis
TIA: Transient ischemic attack
TOF: Time-of-flight
MRI: Magnetic resonance imaging
