# Supplementary material for: The contralateral progression in a cohort of Chinese adult patients with unilateral moyamoya disease after revascularization: a single-center long-term retrospective study
Source: Acta Neurochir (Wien). 2022 Mar 26;164(7):1837–44. doi: 10.1007/s00701-022-05153-6 (PMC9233650; doi:10.1007/s00701-022-05153-6)
Supplement: Supplementary file 1 — Supplementary file1 (PDF 205 KB) [file 701_2022_5153_MOESM1_ESM.pdf]

Patient No.	Age	GENDER	Original Leftesion side	Original 1esion side--DSA grade	Onset（I:Ischemic I, H:HemorrhagicH，T+O:TIA and other)	Hypertension (yes Y, no N)	Aneurysm (yes Y, no N)	Diabets (yes Y, no N)	Other (yes Y, no N)	Family history(yes Y, no N)	Follow-up duration	Process(yes Y, no N)	Process side (Left Left，Right Right)	Process DSA grade	Process symptom (I:Ischemic I, H:HemorrhagicH，T+O:TIA and other)
1	31	M	Left	5	I	Y	N	N	N	N	54	Y	Right	3	I
2	34	F	Right	3	I	N	N	Y	N	N	72	N			
3	37	M	Right	3	I	N	N	N	N	N	54	N			
4	38	M	Right	3	H	N	N	N	N	N	62	Y	Left	3	H
5	39	F	Right	3	T+O	N	N	N	N	N	62	N			
6	39	F	Right	3	I	N	N	N	N	N	78	N			
7	39	F	Right	2	T+O	N	Y	N	N	Y	64	Y	Left	2	T+O
8	39	F	Left	4	T+O	N	N	N	N	Y	75	Y	Left	3	I
9	40	F	Left	2	H	N	N	N	N	N	50	N			
10	40	M	Right	3	I	N	N	N	N	N	60	N			
11	41	F	Right	4	H	N	N	N		Y	58	N			
12	41	M	Left	4	T+O	Y	N	N	N	N	58	N			
13	41	M	Left	2	T+O	N	N	N	N	N	58	N			
14	43	M	Left	3	I	N	N	N	N	N	76	N			
15	44	M	Right	4	T+O	N	N	N	N	N	58	N			
16	44	F	Right	3	H	N	Y	N	N	N	62	N			
17	44	M	Right	4	H	Y	N	N	N	N	65	N			
18	45	M	Right	3	T+O	N	N	N	N	N	49	N			
19	45	F	Right	4	H	N	Y	Y	Y	N	56	N			
20	45	M	Right	3	H	N	N	N	N	N	59	N			
21	45	F	Right	4	T+O	N	N	N	N	Y	70	N			
22	45	F	Left	4	H	N	N	N	N	N	71	N			
23	45	F	Right	3	H	N	N	N	N	N	76	N			
24	46	M	Right	4	I	N	N	N	N	N	55	N			
25	46	F	Left	4	T+O	N	N	N	N	N	57	N			
26	46	F	Right	5	I	N	N	N	N	N	62	N			
27	46	M	Left	5	H	N	N	N	N	N	63	N			
28	46	F	Right	4	T+O	N	N	N	N	N	66	N			
29	46	M	Right	2	H	N	N	N	N	N	67	N			
30	46	F	Right	2	T+O	N	N	N	N	N	69	N			
31	46	M	Left	3	I	N	N	N	N	N	73	N			
32	46	F	Left	4	T+O	N	N	N	N	N	76	Y	Left	3	T+O
33	47	F	Right	5	I	N	N	N	N	N	67	N			
34	47	F	Left	4	T+O	N	N	N	N	N	56	Y	Right	2	I
35	48	F	Right	4	I	N	N	N	N	N	49	N			
36	48	M	Left	3	I	Y	N	Y	N	N	52	N			
37	48	M	Left	3	I	N	N	N	N	N	60	N			
38	49	F	Left	4	I	Y	N	N	N	Y	49	N			
39	49	F	Right	4	4	N	N	N	N	N	67	N			
40	49	F	Left	3	H	Y	Y	Y	Y	N	75	N			
41	49	F	Left	4	T+O	N	N	N	N	N	78	Y	Right	2	T+O
42	50	M	Right	4	H	N	N	N	N	N	52	N			
43	50	F	Left	3	T+O	N	N	N	N	N	54	N			
44	50	M	Left	4	H	N	N	N	N	N	64	N			
45	50	F	Right	5	H	N	N	N	N	N	64	N			
46	50	F	Right	3	4	N	N	N	N	N	75	N			
47	51	F	Right	5	I	N	N	N	N	N	53	N			
48	51	F	Left	4	T+O	N	N	N	N	N	73	N			
49	51	M	Left	3	H	Y	N	N	N	N	77	N			
50	52	F	Left	3	I	N	N	N	N	N	48	N			
51	52	M	Right	4	H	N	N	N	N	N	48	N			
52	52	M	Right	3	H	N	N	N	Y	N	50	N			
53	52	F	Right	4	H	Y	N	N	N	N	53	N			
54	52	M	Left	5	I	N	N	N	N	N	54	N			
55	52	F	Right	3	I	N	N	N	N	N	69	N			
56	52	F	Left	4	H	N	N	N	N	N	70	N			
57	52	M	Right	3	I	N	N	N	N	N	74	N			
58	53	M	Right	2	T+O	N	N	N	N	N	51	N			
59	53	F	Left	4	H	N	N	N	N	Y	53	N			

60	53	F	Left	4	H	Y	N	N	N	N	53	N			
61	53	F	Right	3	H	N	N	N	N	N	53	N			
62	53	F	Right	3	T+O	N	N	N	N	Y	56	N			
63	53	M	Left	3	T+O	Y	N	N	N	N	68	N			
64	53	M	Right	3	T+O	N	N	N	N	N	71	N			
65	53	M	Left	6	4	N	N	N	N	N	71	N			
66	53	M	Left	3	4	N	Y	N	N	N	72	N			
67	53	M	Left	4	I	Y	N	N	Y	N	75	Y	Right	3	I
68	54	M	Left	3	T+O	N	N	N	N	N	56	N			
69	54	F	Left	3	H	N	N	N	N	N	61	N			
70	54	F	Right	4	H	N	N	N	Y	N	67	N			
71	54	F	Left	3	H	N	N	N	N	N	74	N			
72	46	F	Left	3	T+O	N	N	N	N	N	76	Y	Right	3	T+O
73	50	F	Right	3	H	N	N	N	N	Y	68	N			
74	50	F	Right	2	I	N	N	N	N	N	71	N			
75	51	F	Left	3	H	N	N	N	N	N	49	N			
76	51	F	Right	5	4	N	N	N	N	N	57	N			
77	51	M	Right	3	I	N	N	N	N	N	70	N			
78	51	M	Right	3	H	N	N	N	Y	N	75	N			
79	52	F	Right	2	H	N	N	N	Y	N	57	N			
80	52	F	Right	5	I	Y	N	Y	N	N	59	N			
81	52	F	Left	3	H	N	N	N	N	N	61	N			
82	52	M	Right	4	I	N	N	N	N	N	77	N			
83	53	F	Right	4	H	N	N	N	N	N	65	N			
84	53	M	Right	3	I	N	N	N	N	N	67	N			
85	53	F	Right	4	I	N	N	N	N	N	67	N			
86	53	F	Right	4	I	N	N	N	Y	N	70	N			
87	54	F	Left	4	H	N	N	N	N	N	63	N			
88	54	F	Right	4	H	N	Y	N	N	N	65	N			
89	54	M	Right	5	I	N	N	Y	N	Y	76	N			
